# Program Evaluation and Chronic Diseases: Methods, Approaches, and Implications for Public Health

**Published:** 2005-12-15

**Authors:** Leonard Jack, Qaiser Mukhtar, Maurice Martin, Mark Rivera, S. René Lavinghouze, Jan Jernigan, Paul Z Siegel, Gregory Heath, Dara Murphy

**Affiliations:** Applied Behavioral Research, Epidemiology, Surveillance, and Evaluation (ABRESE) Team, Program Development Branch, Division of Diabetes Translation (DDT), National Center for Chronic Disease Prevention and Health Promotion (NCCDPHP), Centers for Disease Control and Prevention (CDC); Dr. Jack is also affiliated with Louisiana State University Health Sciences Center School of Public Health, Behavioral and Community Health Sciences Program, New Orleans, La; ABRESE Team, Program Development Branch, DDT, NCCDPHP, CDC, Atlanta, Ga; ABRESE Team, Program Development Branch, DDT, NCCDPHP, CDC, Atlanta, Ga; ABRESE Team, Program Development Branch, DDT, NCCDPHP, CDC, Atlanta, Ga; Program Services Team, Division of Oral Health, NCCDPHP, CDC, Atlanta, Ga; Cardiovascular Health Branch, NCCDPHP, CDC, Atlanta, Ga; Community Health and Program Services Branch, Division of Adult and Community Health, NCCDPHP, CDC, Atlanta, Ga; Program Branch, Division of Nutrition and Physical Activity, NCCDPHP, CDC, Atlanta, Ga; Program Development Branch, DDT, NCCDPHP, CDC, Atlanta, Ga

## Introduction

As the burden of chronic diseases in the United States continues to increase, greater efforts are being made to identify and implement interventions that successfully reduce disease risk, improve access to high-quality health care, and create sustainable health-promotion programs that ultimately improve health status and quality of life (1). Identifying effective primary and secondary prevention strategies through tailored program evaluation efforts has become an essential public health function in clinical and public health settings (2). Articles in this issue of *Preventing Chronic Disease (PCD) *address various aspects of program evaluation, such as planning, methods, approaches, stakeholder involvement, and the use of program evaluation findings to guide the direction of future programs.

The articles presented in this issue have three primary goals: 1) to provide the reader with practical examples of program evaluation that can be immediately applied in other settings; 2) to carefully discuss the way program missions and objectives, stakeholder interests, evaluation theory, and evaluation methods are considered when conducting, analyzing, and reporting the status of program outcomes; and 3) to candidly explore the use of evaluation frameworks, logic models, and organizational strategic planning to increase capacity for routinely monitoring program outcomes at the national, state, and community levels.

## Chronic Diseases in the United States

Chronic diseases such as diabetes, cardiovascular diseases (particularly heart disease and stroke), and cancer are among the most prevalent and costly of all health problems (3-6). More than 90 million Americans live with chronic diseases (3), and chronic diseases account for three fourths of the nation's $1.4 trillion in medical care costs and one third of the years of potential life lost before age 65 (3). Individual, family, health system, community, and societal factors are all believed to have contributed to the rise in chronic disease rates in the United States (7). Factors postulated to explain this phenomenon range from increased prevalence of individual risk factors (8), a lack of health care resources for the poor and underserved (9), and environmental conditions that do not support the adoption and sustainability of healthy eating and physical activity behavior (10). Collectively, these factors may express themselves differently from one sociogeographical context to another. As a result, a combination of tailored, multifaceted, and multidisciplinary clinical and public health approaches is needed to systematically intervene.

More recent public health discussions about the role of social determinants and health disparities among women and racial and ethnic minorities in the United States help illustrate the complex and dynamic aspects of chronic diseases. The discussions also emphasize the dynamic interactions between individuals and their social and physical environments (11). Addressing the reciprocal relationship between the individual and the environment requires complementary clinical and public health approaches as well as the unique contributions of numerous partners (2). Clearly, reducing the burden of chronic diseases requires amassing and coordinating efforts from various traditional public health partners as well as other untapped resources that share an interest in preventing chronic diseases and improving the quality of life of people with chronic diseases.

## The Centers for Disease Control and Prevention

The Centers for Disease Control and Prevention (CDC) is dedicated to helping Americans live long, healthy, and satisfying lives (12). The organization's missions include preventing death and disability from chronic diseases; promoting maternal, infant, and adolescent health; and promoting healthy personal behaviors. To accomplish these missions, the CDC relies on the strengths and contributions of a diverse group of committed partners such as state and local health departments, international and national organizations, academic institutions, philanthropic foundations, industry and labor groups, professional associations, and volunteer and community organizations (12).

Through its relationship with collaborating partners, the CDC is able to provide national leadership in health promotion and disease prevention by 1) conducting public health surveillance, epidemiologic studies, and behavioral interventions; 2) disseminating guidelines and recommendations for public health interventions; and 3) helping state health departments build their capacity to prevent chronic diseases (12). The CDC is committed to applying research findings to chronic disease prevention and control to improve the health of the people in the United States. To accomplish this goal, the CDC is developing, implementing, and evaluating national, regional, state, and community programs. During this process, the CDC considers the distribution of risk factors among vulnerable populations, social determinants of health, and characteristics of the social and physical environments.

## Program Evaluation: Demand for Accountability and Results

Evaluation of national, regional, state, and community programs remains a priority of the CDC, which uses various tailored program evaluation activities designed to meet their stakeholders’ needs and capacities. In addition, demand is increasing for 1) a formal evaluation infrastructure for regularly assessing the effectiveness of public health programs (13); 2) the creation and maintenance of evaluation monitoring systems to collect, analyze, and interpret public health intervention findings (13,14); 3) the capacity to monitor progress toward improving the health of vulnerable populations (15,16); and 4) evidence that findings about changes in health outcomes (whether positive or negative) are used to make changes in programs (17,18). Using program evaluation activities that incorporate all four of these important factors will better position the CDC and its partners to make critical decisions about program performance and the use of federal funds in a way that demonstrates sound stewardship of taxpayer money.

The demand for accountability is not new to the CDC. In 2002, Milstein et al explained:

With demands for accountability and results at a high level, the CDC faced the problem that many of its programs involved collaborative, multifaceted initiatives with communities across the nation and around the world. Engaging these community partners required complex approaches melding policy, structural, and individual change that were 1) implemented differently in different contexts and 2) hard to measure feasibly and consistently. Furthermore, the ultimate outcomes of interest, such as reductions in hypertension, HIV infection, obesity, or violence, were ones that might take years to materialize. The CDC remained committed to showing that its efforts as an agency were worthwhile. Yet understanding the precise effects of a single program under these circumstances proved to be an extraordinary challenge. (13)

Fortunately, considerable development in program evaluation approaches in the last century has made it possible to embrace the complexities of public health (19). For example, *decision and accountability, utilizations focused, client centered and responsive, case study,* and *outcomes monitoring and value added* are a few of the evaluation approaches that have met the high program evaluation standards of usability, feasibility, propriety, and accuracy (19). Because of the need for multifaceted, multidisciplinary, and multidimensional approaches to address real-world factors that influence chronic diseases, the use of one or more evaluation approaches to ascertain program effectiveness is imperative.

## Program Evaluation and Chronic Diseases

Gathering evidence to demonstrate accountability for program outcomes is a priority for the CDC. Evaluation is one of the 10 essential public health services and is considered a critical function of public health agencies (2). The articles in this program evaluation issue of *PCD* include Original Research, Community Case Studies, Essays, Step-by-Step, Tools & Techniques, and Book Reviews. Through these various types of articles, we hope to expose readers to the value of stakeholder participation at all levels of program design; share a rich discussion of how program evaluation findings can and should be used to make improvements in the implementation and evaluation of existing and future chronic disease programs; promote the identification of program evaluation areas that need additional attention and improvements; and explore examples of evaluation methods and approaches.

## Original Research

In Mukhtar et al's original research article on *Healthy People 2010* diabetes objectives (20), evaluators describe the way the CDC's Division of Diabetes Translation (DDT) adopted and monitored progress toward selected *Healthy People 2010* objectives. These objectives included improving the rates of preventive care service, such as hemoglobin A1c tests and annual foot and eye examinations, among people with diabetes. Data from the Behavioral Risk Factor Surveillance System (BRFSS) diabetes module were used to evaluate progress toward achieving *Healthy People 2010* targets. Evaluators compared 2003 data with *Healthy People 2010* targets and 2000 baseline rates. The degree to which the DDT and its partners achieved the *Healthy People 2010* targets is discussed, as are challenges and important factors to consider when selecting and monitoring these national objectives. Areas for future research and evaluation are also discussed.

In their original research article, Besculides et al describe an evaluation approach that identifies best practices in implementing lifestyle interventions for women in the WISEWOMAN program (21). The authors report using qualitative and quantitative methods, or a *mixed-method approach,* in this evaluation effort. Specifically, they use quantitative program performance data to identify high- and low-performance WISEWOMAN sites and use qualitative interviews, observations, and focus groups to understand underlying strategies for implementing the interventions. The authors conclude with a discussion about the relevance of using a mixed-method approach to conduct evaluation of community-based interventions.

Hypertension is the leading cause of stroke, coronary artery disease, heart attacks, and heart and kidney failure in the United States (22). Programs that provide free or low-cost blood pressure medications and preventive treatment protocols based on authoritative guidelines may not only improve health among patients with hypertension but also result in substantial cost savings. Rein et al found that the state-funded education and direct service program in Georgia resulted in better health outcomes than two other scenarios — no preventive treatment for high blood pressure and the average U.S. private sector preventive treatment (23). Evaluators conclude with a discussion about the need for more evidence-based and cost-effective programs to prevent heart disease and stroke.

## Community Case Studies

In Houston et al's community case study, the authors share with readers the evaluation of South Carolina's Diabetes Prevention and Control Program and the Diabetes Today Advisory Council's effort to conduct a 1-day conference for people with diabetes. The conference offered educational sessions on improving diabetes self-management practices (24). Authors describe the evaluation efforts since the conference's inception and report results from data gathered using qualitative and quantitative evaluation methods. Of importance is a discussion of the evolution of the evaluation planning and methodology as the conference became more sophisticated and far-reaching over a period of years. Using focus groups, a general participant questionnaire, and a Diabetes-Related Understanding Scale, evaluators were able to determine that participants were motivated to adopt diabetes self-management behavioral changes and were pleased with the conference overall. The evaluation also demonstrated that the conference effectively improved diabetes management skills among attendees. Evaluators concluded that the conference could help supplement and reinforce formal diabetes education.

Ideally, program evaluation should be considered at the inception of any public health program. Balamurugan et al explain that the effectiveness of programs in underserved rural areas of Arkansas was impeded because of the lack of advance evaluation planning (25). The authors report that the state health department was successful in establishing 12 diabetes self-management education (DSME) programs in underserved counties that had a disproportionately high prevalence of diabetes. Although some of the barriers faced by the programs were anticipated (e.g., staffing and reimbursement issues), the authors discuss the reasons only some of those barriers could be addressed effectively. Unanticipated barriers were encountered as well, such as inconsistent data collection procedures, a suboptimal data collection capacity, participant retention issues, and the lack of an adopted and implemented evaluation plan among DSME program sites. The authors offer strategies to overcome barriers and use what they learned to plan the new wave of DSME sites that will soon be initiated in similar geographical regions of Arkansas.

## Step-by-Step

In 1999, the CDC published the *Framework for Program Evaluation in Public Health* (26). Martin and Heath use this framework to discuss a hypothetical case study of a physical activity program to prevent diabetes. In their article, the authors discuss each of the six steps: 1) engage stakeholders, 2) describe the program, 3) focus the evaluation design, 4) gather credible evidence, 5) justify conclusions, and 6) ensure use and share lessons learned (27). The authors describe stakeholders and present a logic model with possible short-term, intermediate, and long-term objectives. They briefly discuss quantitative and qualitative data gathering and analysis and conclude with a brief discussion about the ways to share program evaluation findings with the community.

## Essays

An essay by Martin and Thomas addresses Office of Management and Budget (OMB) clearance (28). Federally funded program evaluations usually require collecting data from the public. The data are used to measure processes, impact, and outcomes resulting from health promotion programming. Although collecting these data is important, so is considering the burden of paperwork on the public. Martin and Thomas discuss the process for attaining approval from the OMB for federally sponsored data collection. They also describe how important it is for program evaluators and their collaborators and partners working with the federal government to plan early and consider OMB clearance requirements.

## Tools & Techniques

The *Steps to a HealthierUS* Cooperative Agreement Program (i.e., the Steps Program) focuses on chronic disease prevention and health promotion efforts to reduce the burden of diabetes, obesity, asthma, and related risk factors. In their article, MacDonald et al describe the need for the Steps Program to coordinate national and community evaluation efforts (29). The authors discuss the importance of providing national leadership for evaluation among all Steps Program sites while also allowing flexibility for site-specific evaluation efforts that would allow movement toward well-designed and complementary evaluation plans at national and community levels.

Mukhtar et al (30) describe their experience in developing the Diabetes Indicators and Data Sources Internet Tool (DIDIT). This user-friendly Web-based tool contains information on 38 diabetes indicators and their associated data sources. The DIDIT was developed in collaboration with multiple stakeholders, including state representatives, the CDC, and contractors. The authors highlight the elements that were essential for the tool's development. Expertise in diabetes surveillance and software development as well as stakeholder enthusiasm and dedication were important components. These components were complemented by the project leader's strong leadership skills and sense of vision, clear communication and collaboration among all team members, and commitment from the management of the Division of Diabetes Translation.

In their article, Tucker et al begin with a brief history of the Racial and Ethnic Approaches to Community Health (REACH 2010) initiative (31). Authors discuss the way 40 REACH 2010 communities (African American, Alaska Native, American Indian, Asian American, Hispanic, and Pacific Islander communities) use community-based participatory approaches to reduce risk factors for and the prevalence of chronic diseases. Using a logic model, the authors describe the way program activities are related to program theory as well as short- and long-term program outcomes. The article emphasizes the need to conduct local, site-specific evaluations as well as a national evaluation that takes into consideration cross-site assessment of successful partnerships. The authors discuss the way using qualitative data collected from REACH 2010 projects with a management information system called the *REACH Information Network* will help users understand how program components influence system changes. The authors also describe the way quantitative data are systematically collected using the REACH Risk Factor Survey to establish estimates of program effects. Local, site-specific, and national evaluations using qualitative and quantitative evaluation methods will help determine whether local interventions decrease health disparities.

## Book Review

This issue includes Lavinghouze's book review (32) of *Practical Program Evaluation Assessing and Improving Planning, Implementation, and Effectiveness* by Huey-Tsyh Chen (33). Lavinghouze describes Chen's efforts to provide a program evaluation taxonomy that would be particularly useful to individuals new to the field of program evaluation as well as to more seasoned evaluators who want to encourage stakeholder understanding of evaluation. She describes Chen's ability to provide a thorough overview and review of the theory-driven approach to evaluation and apply it to the taxonomy he presents. She points out that although terms and definitions used in the book are inconsistent with those found in current literature, Chen encourages the readers to broaden their perspectives so that they can embrace this new terminology. According to Lavinghouze, Chen's book emphasizes acknowledging the stakeholder throughout the evaluation process. She concludes that Chen's taxonomy is a major step in the overall conceptualization of the evaluation process and that the taxonomy enhances evaluators' attempts to understand and appropriately apply evaluation designs at a practical program level.

## Conclusion

The diverse nature of evaluation efforts undertaken by the CDC and its many partners highlights the interest and commitment to designing, implementing, and evaluating high-quality chronic disease prevention and control activities that are responsive to target audience and stakeholder needs. The use of evaluation is being integrated into the accountability movement and is embedded in a consumer-oriented public health ideology (34). It is becoming an increasingly important accountability tool in the current environment and is considered a necessary component of decision making about the use of federal funds to support successful programs. According to Segerholm, "Against this background, it is high time to start critically examining evaluation itself as a phenomenon and practice" (34). We hope the articles in this issue not only emphasize the importance of program evaluation but also provide our readers with examples to incorporate into evaluation approaches, stakeholder engagement strategies, and their own public health efforts.
